# Continuous Glucose Monitoring Beyond Intensive Insulin Therapy in Type 2 Diabetes: Evidence, Over-the-Counter Devices, Equity, and Clinical Implementation

**DOI:** 10.7759/cureus.112200

**Published:** 2026-07-07

**Authors:** Andrzej Bazan, Oliwer Plichta, Natalia Radzinska, Klaudia Kalinowska

**Affiliations:** 1 General Medicine, St. Anthony Hospital, Ząbkowice Śląskie, POL; 2 Medicine, University Clinical Hospital Jan Mikulicz-Radecki, Wroclaw, POL; 3 Midwifery, Faculty of Health Sciences, Wroclaw Medical University, Wroclaw, POL; 4 Primary Care, Samodzielny Publiczny Zakład Opieki Zdrowotnej Ministerstwa Spraw Wewnętrznych i Administracji (SP ZOZ MSWiA), Opole, POL

**Keywords:** ambulatory glucose profile, basal insulin, cgm, continuous glucose monitoring, diabetes technology, health equity, primary care implementation, type 2 diabetes

## Abstract

Continuous glucose monitoring (CGM) measures interstitial glucose over time and is increasingly used beyond intensive insulin therapy in type 2 diabetes. In routine care, CGM is useful when pattern information can improve management, not simply because more glucose data are available. This structured narrative review summarizes evidence, device categories, over-the-counter (OTC) systems, equity considerations, and practical implementation for adults with type 2 diabetes who are not receiving intensive insulin therapy, with particular focus on non-insulin-treated adults and those treated with basal insulin without prandial insulin. In non-insulin-treated adults, CGM may help identify fasting or postprandial hyperglycemia, support lifestyle and adherence discussions, and clarify discordance between hemoglobin A1c (HbA1c) and daily glucose patterns, although evidence remains stronger for glycemic metrics than for long-term outcomes. In adults treated with basal insulin without prandial insulin, randomized trial evidence supports CGM for glycemic assessment and safer treatment review. Systems cleared by the US Food and Drug Administration (FDA) for OTC CGM use and consumer-facing glucose biosensors may increase access to glucose feedback, but intended use, diabetes-specific clinical evidence, and broader wellness use should be distinguished. This review presents a practical implementation framework that begins with patient selection, followed by education, structured interpretation of CGM data, and integration of findings into lifestyle counselling, medication review, and shared clinical decision-making while accounting for coverage, digital access, language-sensitive support, and safety risks. CGM should function as structured decision support, not passive surveillance or consumer data collection.

## Introduction and background

Continuous glucose monitoring (CGM) refers to the sensor-based measurement of interstitial glucose with the repeated display of glucose values, trends, and summary metrics [1,2]. Although CGM was first established most firmly in intensive insulin therapy, current guidance and evidence have extended its relevance to selected adults with type 2 diabetes who are not receiving intensive insulin therapy, particularly those treated without insulin or with basal insulin alone rather than multiple daily insulin injections, insulin pump therapy, or automated insulin delivery systems [1,3-11]. In these patients, the main question is not complex insulin dosing but whether glucose patterns can improve decisions that hemoglobin A1c (HbA1c) and occasional capillary glucose testing cannot answer well.

For the purposes of this review, "beyond intensive insulin therapy" refers primarily to adults with type 2 diabetes treated without insulin or with basal insulin alone. Adults treated only with non-insulin glucose-lowering therapies, including glucagon-like peptide-1 receptor agonists (GLP-1 RAs) or sodium-glucose cotransporter-2 (SGLT2) inhibitors, are included within the non-insulin-treated group. Premixed insulin and other mixed regimens are discussed only where directly relevant to available evidence.

CGM may clarify fasting or postprandial hyperglycemia, reveal unrecognized hypoglycemia, explain discordance between HbA1c and symptoms, guide basal insulin review, or make lifestyle and medication discussions more specific. Evidence syntheses and selected trials support improvements in glycemic metrics in selected non-insulin-treated and basal insulin-treated adults [5-12]. The evidence remains stronger for short- to medium-term glycemic outcomes than for long-term complications, healthcare utilization, cost-effectiveness, or routine continuous use.

The clinical context has also changed with systems cleared by the US Food and Drug Administration (FDA) for over-the-counter (OTC) CGM use in specified adult non-insulin-using populations [13-15]. OTC access may allow more patients to encounter sensor data outside prescription and coverage pathways, but it also increases the need to distinguish regulatory clearance, wellness use, and clinical benefit. Coverage, cost, language access, digital readiness, and workflow determine whether sensor data become useful care or a burden [16-19]. Safeguards against unsupervised medical action and unregulated consumer glucose claims are also needed as CGM becomes more accessible [13-15,20].

This review followed a structured narrative design rather than a systematic review design. Targeted PubMed searches and source verification were updated through March 31, 2026, with supplementary checks of official diabetes society, FDA, Centers for Medicare and Medicaid Services (CMS), and publisher sources to confirm current guidance, device clearance documents, coverage policy, safety communications, and article details. Searches combined terms related to CGM technology, treatment intensity, device categories, glycemic metrics, implementation, OTC systems, coverage, and equity, including "continuous glucose monitoring", "CGM", "type 2 diabetes", "non-insulin-treated type 2 diabetes", "basal insulin", "professional CGM", "personal CGM", "real-time CGM", "intermittently scanned CGM", "over-the-counter CGM", "time in range", "ambulatory glucose profile", "Stelo", "Libre Rio", "Lingo", "Medicare coverage", "health equity", and "language disparities".

Source selection prioritized current clinical guidance, CGM metric consensus recommendations, systematic reviews, meta-analyses, FDA and CMS documents, access and equity studies, and selected primary studies when higher-level evidence did not adequately address a specific clinical use case. Neutral and negative findings were considered when reported in included evidence syntheses or selected clinical studies. The review focused on sources available through March 31, 2026, while retaining older foundational sources when needed for CGM metric interpretation or established trial evidence. Sources were included when they supported the discussion of non-insulin-treated or basal insulin-treated type 2 diabetes, FDA-cleared OTC CGM status, patient selection, clinical interpretation, access, equity, safety, or routine implementation. No formal systematic review methods, Preferred Reporting Items for Systematic Reviews and Meta-Analyses (PRISMA) screening, study-level risk-of-bias assessment, or quantitative synthesis were applied.

## Review

CGM use beyond intensive insulin therapy can be organized around a question-driven pathway: identify the management problem, select the least burdensome approach, interpret the pattern with standard metrics, and link the finding to education, pharmacotherapy review, safety counselling, or follow-up.

Current CGM terminology and device categories

CGM should not be treated as one uniform intervention. Professional CGM used to clarify postprandial hyperglycemia, prescription real-time CGM used with basal insulin, and OTC glucose biosensor use for lifestyle feedback represent different clinical situations. They differ in data visibility, alerts, duration, patient involvement, clinician oversight, and clinical purpose [1,10,13-15].

In this review, personal CGM is used as a clinical descriptor for patient-used CGM worn outside the clinic, rather than as a separate regulatory category. Professional CGM is supplied through a clinical practice for a limited assessment period and may be blinded or unblinded. Real-time CGM automatically displays or transmits values and trends, often with alerts; intermittently scanned CGM requires the user to scan the sensor. Real-time systems may be more useful when alerts or nocturnal hypoglycemia warnings are important, while intermittently scanned systems may be sufficient for structured pattern review or short-term behavior assessment [1,5-10].

Prescription status and device function are separate issues. Prescription CGM is obtained through a clinician's order and is usually embedded in training, interpretation, and follow-up. OTC CGM can be obtained without a prescription, but it is not appropriate for every clinical purpose and should not prompt treatment changes without professional input. For diabetes-focused clinical discussion, the OTC systems most relevant to this review are Stelo and Libre Rio, both directed toward adults aged 18 years or older who are not using insulin or are non-insulin users [13,14]. Lingo is also an FDA-cleared OTC integrated CGM for adults aged 18 years or older not using insulin, but its cleared language is oriented toward glucose awareness and lifestyle or behavior feedback rather than diabetes treatment decisions; it is therefore discussed separately as a consumer-facing glucose biosensor [15].

CGM interpretation depends on summary metrics and recurring patterns. Time in range describes the proportion of sensor readings within a target range, while time above range and time below range describe hyperglycemia and hypoglycemia exposure. For most nonpregnant adults with diabetes, international consensus recommendations commonly use 70-180 mg/dL (3.9-10 mmol/L) as the target range, with goals of more than 70% time in range, less than 25% time above 180 mg/dL (>10 mmol/L), less than 5% time above 250 mg/dL (>13.9 mmol/L), less than 4% time below 70 mg/dL (<3.9 mmol/L), and less than 1% time below 54 mg/dL (<3 mmol/L), individualized according to age, comorbidity, treatment regimen, and hypoglycemia risk [2,3]. The glucose management indicator estimates an HbA1c-like value from the mean CGM glucose, and the ambulatory glucose profile displays recurring patterns. These metrics complement HbA1c but do not replace symptoms, medication review, or clinical judgment [1-3,10].

Clinical evidence beyond intensive insulin therapy

CGM evidence in type 2 diabetes should be interpreted according to treatment intensity and clinical purpose. A patient using multiple daily insulin injections, a patient using basal insulin alone, and a patient treated without insulin do not face the same risks or decisions. In non-intensive type 2 diabetes care, CGM is best understood as a tool for pattern recognition, treatment review, hypoglycemia detection in higher-risk regimens, and structured feedback rather than as a universal monitoring requirement.

Recent evidence syntheses support modest glycemic benefits from CGM in selected adults with type 2 diabetes, but the magnitude varies by baseline HbA1c, treatment regimen, device type, intervention duration, and educational support. In a meta-analysis of randomized trials in adults with type 2 diabetes, CGM was associated with an HbA1c mean difference of -3.43 mmol/mol, equivalent to -0.31 percentage points, compared with self-monitoring of blood glucose [5]. In a meta-analysis limited to non-insulin-treated type 2 diabetes, CGM was associated with a -0.31 percentage point HbA1c reduction and an 8.63 percentage point increase in time in range [8]. These outcomes allow interpretation beyond average glycemia by showing recurring daily patterns, but most studies remain stronger for short- to medium-term glycemic endpoints than for complications, hospitalization, mortality, or cost-effectiveness.

In non-insulin-treated type 2 diabetes, CGM is most relevant when the clinical question involves patterns that HbA1c cannot show. Examples include whether hyperglycemia is mainly fasting or postprandial, whether apparently acceptable fasting values mask late-day hyperglycemia, whether adherence or meal timing is linked to excursions, or whether structured feedback may help the patient understand daily glucose responses. Meta-analyses focused on non-insulin-treated adults support improved HbA1c and CGM-derived metrics in selected patients, with some evidence for improved treatment satisfaction [8,9]. These findings support selective use, especially with education or a defined management question; they do not support routine continuous CGM for every non-insulin-treated adult.

Behavioral evidence should be interpreted cautiously. In the randomized open-label study by Choe et al., intermittently scanned CGM plus structured education focused on postprandial responses improved HbA1c and self-care-related outcomes versus standard care [12]. This single trial supports the use of CGM as part of a structured educational intervention, but it should not be generalized to passive sensor wear, all CGM modalities, or all adults with type 2 diabetes.

Basal insulin-treated type 2 diabetes is a separate clinical category. These patients are still outside intensive insulin therapy, but insulin exposure makes hypoglycemia, overnight trends, fasting glucose, and titration decisions more important. The randomized clinical trial by Martens et al. studied adults with poorly controlled type 2 diabetes treated with basal insulin without prandial insulin in primary care. Compared with blood glucose meter monitoring over eight months, CGM was associated with greater HbA1c improvement, higher time in the 70-180 mg/dL range, less time above 250 mg/dL, and lower mean glucose [11]. This supports a clearer role for CGM in basal insulin-treated adults, especially when distinguishing fasting hyperglycemia from postprandial excursions, nocturnal hypoglycemia, or over-titration.

OTC CGM and consumer-facing glucose biosensors

OTC CGM has changed how adults with type 2 diabetes may first encounter glucose sensor data. Earlier CGM use was usually linked to prescription, insurance, training, and follow-up. OTC access may lower some entry barriers, especially for adults not using insulin, but easier access does not by itself provide appropriate selection, interpretation, or safe action. OTC CGM is best viewed as a new access route for glucose information that still requires clinical context.

The FDA-cleared OTC systems most relevant to clinical type 2 diabetes care in this review are Stelo and Libre Rio [13,14]. Lingo is retained in the discussion because clinicians may encounter its data in practice, but it is handled separately as a consumer-facing glucose biosensor for adults aged 18 years or older not using insulin and for glucose awareness or lifestyle feedback rather than diabetes treatment decisions [15]. FDA-cleared intended use, clinical evidence, and real-world consumer use should therefore be considered separately.

OTC CGM should not be viewed as equivalent to prescription CGM simply because both use sensor-based glucose measurement. Prescription CGM is usually embedded in eligibility assessment, training, interpretation, and follow-up. Professional CGM is clinician-directed and time-limited; OTC CGM may be purchased and used without those steps. A basal insulin-treated patient using prescription real-time CGM for hypoglycemia risk differs from a non-insulin-treated adult using an OTC sensor to understand postprandial patterns.

The most reasonable clinical use of OTC CGM is question-driven. In a non-insulin-treated adult, short-term OTC CGM may help clarify whether hyperglycemia is fasting, postprandial, late-day, or related to meal timing, activity, adherence, or sleep. Its value is lower without a clinical question, education, or a plan for review.

Clinical interpretation and wellness feedback should be separated. In this review, clinical interpretation refers to CGM data used in the care of a person with diabetes to evaluate glycemic patterns, hypoglycemia risk, medication effects, discordance with HbA1c, or treatment decisions. Wellness feedback refers to glucose pattern awareness used to understand responses to meals, activity, sleep, or daily routines without making diagnostic or medication decisions. Glucose rises after meals are expected, and isolated excursions should not be interpreted as treatment failure or a reason for immediate medication change. OTC CGM data in adults with type 2 diabetes should be interpreted alongside HbA1c, symptoms, medications, comorbidities, hypoglycemia risk, and individualized goals. Evidence supporting CGM in non-insulin-treated type 2 diabetes comes mainly from clinical studies of CGM use with defined interventions and follow-up, not from trials showing that OTC CGM by itself improves clinical outcomes. In people using insulin, sulfonylureas, or other hypoglycemia-prone therapies, unsupervised responses to sensor readings may be unsafe. OTC availability may improve access for some adults, but it is not an equity solution by itself, particularly when education and follow-up are limited [8,9,13-15,18,19].

Safety boundaries, overuse, and inappropriate treatment decisions

CGM can improve safety when it reveals patterns that HbA1c or occasional capillary testing may miss, but the same data can create risk when interpreted without symptoms, medication context, or follow-up. CGM values are clinical information, not automatic instructions. A high or low reading should be interpreted with meals, activity, medication use, illness, alcohol intake, sleep, symptoms, and the overall pattern. Unexpected readings, rapid change, suspected malfunction, or values that do not match symptoms should prompt caution and, when appropriate, capillary confirmation [1-3].

Medication safety is the most important practical concern. Patients using insulin, sulfonylureas, or other hypoglycemia-prone therapies should not change doses solely on the basis of unreviewed CGM patterns unless they have a clinician-directed plan. Overcorrection, omitted medication, unnecessary carbohydrate restriction, or reactive dosing can create harm. Conversely, average glucose or glucose management indicator may look acceptable while recurrent time below range, nocturnal hypoglycemia, or wide glycemic variability remains clinically important [3,4].

Time below range should be treated as a safety signal before hyperglycemia is addressed. Recurrent overnight lows, lows between meals, or lows after physical activity should prompt review of medication intensity, meal timing, alcohol intake, renal function where relevant, and hypoglycemia awareness. Risk of unsafe interpretation or response may be higher in patients using insulin or sulfonylureas and those with impaired hypoglycemia awareness, cognitive impairment, limited health literacy or digital literacy, limited language access, visual or dexterity limitations, or limited support at home. In these patients, recurrent low glucose should prompt the reassessment of glycemic goals and hypoglycemia-prone therapy before further focus on hyperglycemia [2,3,16-19].

Overuse is a related risk. Some patients may check repeatedly, focus on minor fluctuations, or interpret normal postprandial rises as harmful. This may lead to unnecessary restriction, anxiety, or loss of confidence. Clinicians should explain before CGM is started that the aim is to identify recurring patterns, not to eliminate all glucose variation. A short CGM recording should also not override persistent symptoms, a markedly abnormal HbA1c, suspected hypoglycemia, or concern about medication safety.

OTC CGM requires clear safety instructions because it may be used without prescription, training, or planned follow-up. FDA documents for some OTC systems state that users are not intended to take medical action based on device output without consultation with a qualified healthcare professional [13-15]. The selected bibliography did not identify dedicated real-world safety studies evaluating OTC CGM use in higher-risk groups, including insulin or sulfonylurea users, patients with cognitive impairment, those with limited health literacy or digital literacy, or patients with limited language access. Therefore, clinical precautions should be based on the device's intended-use language, hypoglycemia-risk principles, and the patient's ability to interpret and act on data safely. FDA-cleared CGM systems should also be distinguished from consumer devices that claim to measure glucose independently. The FDA has warned against smartwatches and smart rings that claim to measure blood glucose without piercing the skin and has stated that it has not authorized, cleared, or approved such devices to measure or estimate glucose values on their own [20].

Patient selection and initial CGM assessment

CGM use beyond intensive insulin therapy should be selective rather than device-driven. Many adults with type 2 diabetes can be managed appropriately with HbA1c, symptoms, medication review, and occasional capillary glucose testing when clinically indicated. CGM is most useful when usual measures do not explain glycemic patterns, when timing matters more than average glycemia, or when sensor feedback can support a specific behavioral or therapeutic change.

CGM is most actionable in defined clinical situations. In non-insulin-treated type 2 diabetes, CGM may help when HbA1c remains above an individualized target, especially if the clinician needs to determine whether hyperglycemia is fasting, postprandial, late-day, or related to missed medication, inactivity, or meal timing. In basal insulin-treated type 2 diabetes, CGM may identify overnight patterns, fasting trends, unrecognized hypoglycemia, or postprandial excursions that should not trigger further basal insulin titration alone. CGM may also be considered when hypoglycemia is suspected, when HbA1c conflicts with symptoms or home glucose data, or when a motivated patient seeks structured feedback [1,3-12].

The initial assessment should define the treatment context before the device is chosen. The clinician should review HbA1c trajectory, medications, hypoglycemia history, symptoms, relevant comorbidities, recent treatment changes, steroid exposure, meal patterns, activity, work schedule, and prior monitoring experience. Device choice should match the purpose. Professional CGM may be sufficient for short-term clarification; prescription personal CGM may fit continued feedback, alerts, or medication adjustment; and OTC CGM may suit selected non-insulin-using adults who want structured feedback, provided education and interpretation are planned.

Practical readiness is part of selection. Before recommending CGM, the clinician or clinical team should determine whether the patient can obtain the device, afford sensors if repeat or continued use is planned, use a reader or smartphone, understand the display, respond appropriately to unexpected readings, and bring or share data for review. Language needs, health literacy, digital confidence, visual or dexterity limitations, and family or caregiver support should be considered [16-19]. If these supports are limited, professional CGM, diabetes education, medication review, or delayed sensor use may be safer.

Table 1 summarizes the practical clinical scenarios in which CGM may be considered beyond intensive insulin therapy.

**Table 1:** Clinical use of CGM beyond intensive insulin therapy in type 2 diabetes CGM: continuous glucose monitoring; FDA: Food and Drug Administration; GMI: glucose management indicator; HbA1c: hemoglobin A1c; OTC: over-the-counter

Clinical scenario	Potential rationale for CGM	Possible CGM approach	Interpretation points	Cautions or limitations
Non-insulin-treated type 2 diabetes with HbA1c above an individualized target	Clarify whether hyperglycemia is fasting, postprandial, late-day, or linked to adherence, meals, or activity	Short-term personal CGM, professional CGM, or selected OTC CGM with follow-up	Review time above range, postprandial patterns, fasting trends, and relation to daily routines	Evidence supports selected use and glycemic metrics; not routine continuous CGM for all
Basal insulin-treated type 2 diabetes without prandial insulin	Support safer treatment review and distinguish fasting hyperglycemia from postprandial excursions or nocturnal hypoglycemia	Prescription personal CGM or professional CGM; real-time CGM may be useful when hypoglycemia concern is present	Review overnight trends, fasting glucose, time below range, time above range, and mean glucose	Do not escalate basal insulin if fasting or overnight glucose is already near target
Suspected hypoglycemia or hypoglycemia-prone therapy	Detect unrecognized lows, especially overnight or between meals	Personal CGM or professional CGM depending on urgency and need for alerts	Prioritize time below range, timing of lows, symptoms, medication context, and confirmatory testing when needed	Medication changes should be clinician-guided, especially with insulin or sulfonylureas
Discordant HbA1c, symptoms, or capillary readings	Clarify whether HbA1c matches usual glycemia and identify hidden variability or excursions	Professional CGM or short-term personal CGM	Compare CGM patterns, GMI, HbA1c, symptoms, recent treatment changes, and comorbid factors	GMI is not interchangeable with HbA1c; discordance requires clinical review
Lifestyle or nutrition counselling	Make feedback more specific by linking recurrent patterns to meals, activity, alcohol, sleep, or routines	Intermittent personal CGM, professional CGM, or selected OTC CGM	Focus on recurrent, prolonged, actionable patterns rather than isolated excursions	Avoid food anxiety, unnecessary restriction, and overinterpretation of normal postprandial rises
Medication intensification or de-intensification	Support treatment decisions by identifying the dominant glycemic pattern or hypoglycemia risk	Personal or professional CGM with planned clinician review	Link fasting, postprandial, overnight, and time-below-range patterns to medication review	CGM should inform, not replace, standard pharmacologic guidance and clinical judgment
Motivated patient seeking structured feedback	Improve engagement and shared decision-making when the patient can use data constructively	Short-term personal CGM or OTC CGM with education and review	Agree on one or two questions before wear and one or two actions after review	Data without education may add anxiety or burden rather than benefit
OTC CGM use without established clinical follow-up	Provide a route to glucose awareness outside prescription pathways	OTC CGM with clinician review when data influence diabetes care	Ask why the device was used, what actions were taken, and whether readings match symptoms	FDA-cleared OTC status is not proof of benefit for every use; avoid unsupervised medication changes

Interpreting CGM data in clinical practice

CGM reports should be interpreted through a sequence rather than as isolated graphs. The first step is to define the context: why the sensor was used, whether it was personal, professional, prescription, or OTC, how long it was worn, and what treatment the patient was using. Illness, travel, steroid exposure, medication changes, altered diet, exercise, and inconsistent wear should be reviewed before conclusions are drawn.

The second step is to decide whether the report is adequate for the clinical question being asked. International consensus recommendations commonly use approximately 14 days of CGM data with at least 70% active sensor data to support a stable assessment of usual ambulatory glucose patterns [2]. These criteria should not be applied mechanically to every real-time, intermittently scanned, or professional CGM use case. Shorter professional CGM recordings may still answer focused clinical questions, but they should be interpreted as pragmatic snapshots rather than complete representations of usual glucose exposure.

The ambulatory glucose profile is usually the most useful starting point after data adequacy is checked. Clinicians should review average glucose or glucose management indicator, time in range, time above range, time below range, glycemic variability, and the daily profile. For many nonpregnant adults with diabetes, the commonly used target range is 70-180 mg/dL (3.9-10 mmol/L). Consensus targets often aim for more than 70% time in range, less than 25% time above 180 mg/dL (>10 mmol/L), less than 5% time above 250 mg/dL (>13.9 mmol/L), less than 4% time below 70 mg/dL (<3.9 mmol/L), and less than 1% time below 54 mg/dL (<3 mmol/L) [2,3]. These values are reference points, not automatic treatment rules, and may need individualization in older adults, people with frailty, and patients at high risk of hypoglycemia.

Safety should be reviewed before hyperglycemia patterns. Time below range is particularly important in patients using insulin, sulfonylureas, or other hypoglycemia-prone therapies. Recurrent nocturnal lows, lows between meals, or lows after physical activity should prompt medication review and hypoglycemia education. When symptoms and CGM readings do not match, confirmatory capillary glucose testing may be needed [1-3].

After hypoglycemia risk is assessed, the clinician should identify the dominant hyperglycemia pattern. Persistent fasting or overnight hyperglycemia suggests a different problem from recurrent postprandial excursions or late-day hyperglycemia. In a basal insulin-treated patient, this distinction helps prevent treatment changes that address the wrong pattern. In a non-insulin-treated adult, the same pattern review may guide nutrition counselling, adherence review, physical activity advice, or pharmacotherapy intensification [4,10,11]. The glucose management indicator can help translate the mean CGM glucose into an HbA1c-like estimate, but it should not be treated as interchangeable with laboratory HbA1c [2,3].

A practical CGM interpretation pathway can be summarized in five steps: confirm the monitoring context, check data adequacy, look first for hypoglycemia risk, identify the dominant hyperglycemia pattern, and link the pattern to a safe action. This sequence is intended to support clinical review, not replace individualized assessment. If the report does not answer a clinical question, the next step may be education, repeat monitoring, a different monitoring strategy, or no immediate change.

Translating CGM patterns into lifestyle, pharmacotherapy, and shared decisions

CGM can support clinical decisions, but the level of evidence behind each decision should be clear. Guidelines and consensus recommendations support individualized glycemic goals, attention to hypoglycemia, and use of CGM metrics in a clinical context [1-4]. Trial evidence supports selected uses, including CGM in basal insulin-treated type 2 diabetes and intermittently scanned CGM paired with structured education [11,12]. By contrast, many pattern-to-action decisions in routine care, including linking a repeated postprandial excursion to meal timing or medication review, are practical clinical synthesis rather than validated CGM-specific treatment algorithms.

Lifestyle counselling is one of the clearest uses of CGM beyond intensive insulin therapy. HbA1c summarizes average glycemia, but it cannot show whether hyperglycemia occurs after breakfast, after evening meals, during sedentary periods, or after missed medication. CGM can turn general advice into a more specific discussion of repeated breakfast excursions, late-evening hyperglycemia, or post-meal walking [10,12]. Nutrition feedback should be framed carefully because a rise in glucose after eating is expected. The useful question is whether the pattern is recurrent, prolonged, and relevant to the patient's goals. Poor framing may promote food anxiety or unnecessary restriction.

Pharmacotherapy review should be guided by standard diabetes treatment principles, with CGM used to clarify the dominant glucose pattern rather than to dictate a specific drug choice. In non-insulin-treated type 2 diabetes, persistent hyperglycemia despite current therapy may support treatment intensification according to established pharmacologic guidance [4]. If fasting glucose is near target but postprandial hyperglycemia is recurrent, CGM may prompt review of meal patterns, adherence, medication timing, or treatment options that address the observed pattern. If glucose is frequently below range, especially with insulin or sulfonylurea use, the priority should shift toward safety, medication review, and possible de-intensification [3,4,10].

Basal insulin-treated type 2 diabetes requires particular caution. Trial evidence supports CGM as a tool for improving glycemic assessment in adults using basal insulin without prandial insulin [11]. In practice, CGM can help determine whether an elevated HbA1c is driven by fasting hyperglycemia, overnight patterns, postprandial excursions, or glycemic variability. Further basal insulin titration may be inappropriate when fasting or overnight glucose is already near target and the main problem is postprandial hyperglycemia. This is a clinical interpretation of the pattern, not a substitute for individualized medication review. Medication changes should remain clinician-guided and should account for symptoms, hypoglycemia risk, comorbidities, and treatment goals [3,4,11].

CGM can also make adherence and timing problems easier to discuss. Recurrent hyperglycemia after missed doses, delayed meals, late-night eating, steroid exposure, or irregular work schedules can be framed as modifiable patterns rather than personal failure. Most visits should focus on one or two priorities rather than every abnormal metric. The next step might be a meal-timing change, post-meal walking, medication-timing review, basal insulin adjustment, de-intensification of hypoglycemia-prone therapy, diabetes education, repeat CGM, or no medication change. The study by Choe et al. supports this structured approach because intermittently scanned CGM was paired with education rather than passive sensor wear [12].

Access, coverage, and equity as implementation conditions

CGM implementation depends on more than identifying an appropriate clinical indication. A patient may have a reasonable reason to use CGM and still be unable to obtain the device, afford sensors, use the reader or smartphone, share data, or return for interpretation. This matters beyond intensive insulin therapy because the benefit often depends on education, pattern review, and follow-up. Access should be assessed before CGM is recommended.

Coverage is often the first practical barrier. Under the relevant Medicare coverage pathway, CGM coverage requires diabetes mellitus, sufficient training in device use, prescription in accordance with the device's FDA-indicated use, and either insulin treatment or documented problematic hypoglycemia. The policy also requires a recent clinician visit before ordering and continued follow-up [17]. These criteria create a route to covered CGM for many insulin-treated adults and selected patients with problematic hypoglycemia, but not for all non-insulin-treated adults interested in pattern recognition or lifestyle feedback. Medicare criteria should not be treated as a proxy for all US access.

Equity concerns are visible in prescribing patterns. In a national network of federally qualified health centers, CGM prescription orders were uncommon among adults with type 2 diabetes, and lower odds of prescription were observed among patients reporting Hispanic ethnicity, Black race, or uninsured status [18]. In a large integrated health system study, patients with a non-English language preference had lower odds of receiving a CGM prescription than patients with an English language preference [19]. For CGM, language barriers do not end at prescription: patients need understandable instruction on sensor placement, app or reader use, alerts, data sharing, confirmatory testing, and when to contact the clinical team.

Digital readiness should be assessed before CGM is prescribed, recommended, or interpreted. The clinical team should determine whether the patient can use the reader or smartphone, navigate the display, respond appropriately to alerts, upload or bring data, and understand that readings require context. Visual, dexterity, cognitive, literacy, internet, phone access, or support barriers should prompt adaptation rather than exclusion.

Clinic workflow is part of equity. A practice that can prescribe CGM but cannot retrieve reports, review the ambulatory glucose profile, explain results in the patient's preferred language, or act on hypoglycemia patterns may widen disparities rather than reduce them. Clinics should define who checks access, teaches basic use, downloads or reviews data, communicates results, and responds to important findings. Diabetes educators, pharmacists, dietitians, nurses, medical assistants, and interpreters may make CGM feasible outside specialized diabetes clinics [10,16,18,19]. OTC CGM should be evaluated through the same equity lens: it may improve access for some adults, but clinically meaningful access still requires education, data review, and a safe plan for action.

Practical clinical implementation in primary care and endocrinology

CGM workflows must be simple enough for routine care. The process should begin by defining what the sensor is expected to clarify. The question may be diagnostic, therapeutic, or behavioral: suspected nocturnal hypoglycemia, postprandial hyperglycemia, basal insulin over-titration, or more specific nutrition, activity, or medication-timing counselling. When the question is unclear, CGM is more likely to add data than improve care.

The next step is to choose the least burdensome approach that can answer the question. Professional CGM may be appropriate when the clinician needs short-term pattern assessment and the patient does not need continued personal monitoring. Prescription personal CGM may be more appropriate when continued data, alerts, repeated review, or medication adjustment are expected to change management, especially in basal insulin-treated patients or those at higher risk of hypoglycemia. OTC CGM may be reasonable for selected non-insulin-using adults seeking structured feedback, but it should be paired with education and a plan for interpretation rather than treated as a stand-alone consumer product.

Before recommending CGM, the clinician or clinical team should confirm that the patient can obtain, use, and return data from the device. This includes coverage or cost, reader or smartphone access, language needs, health literacy, visual or dexterity limitations, internet access, and family or caregiver support. A patient who cannot upload data from home may still benefit if the practice can review the report in the clinic or use a reader-based or professional system [16-19].

Patient education should be brief, specific, and tied to the reason for monitoring. Patients should understand that CGM measures interstitial glucose, that patterns are usually more useful than isolated values, and that unexpected readings or medication changes require a clear plan.

The first data review should be scheduled rather than left open-ended. A practical review starts with the ambulatory glucose profile and a few metrics: average glucose or glucose management indicator, time in range, time above range, time below range, glycemic variability, and recurring patterns [2,10]. The visit should not become a line-by-line review of sensor readings. The most useful review identifies one or two important patterns and links them to a decision the patient understands.

The action plan should be narrow enough to be carried out. Possible next steps include focused nutrition counselling, medication-timing change, review of missed doses, basal insulin adjustment, de-intensification of hypoglycemia-prone therapy, diabetes education, or repeat CGM. If the report does not show an actionable pattern, no medication change may be the safest conclusion.

Practices should define team roles before CGM use expands. One team member may check access, another may provide setup education, and another may help retrieve or print the report. Clinicians, diabetes educators, pharmacists, dietitians, nurses, medical assistants, and interpreters can each support different parts of the pathway. Endocrinology involvement is most useful when interpretation is complex, hypoglycemia is recurrent, insulin decisions are uncertain, or CGM data do not match HbA1c, symptoms, or the clinical picture [10,16].

A practical CGM workflow can be simplified as follows: define the question, select the least burdensome monitoring approach, prepare the patient, review the main pattern, link it to one or two actions, document the plan, and reassess whether continued CGM is needed. This sequence should be understood as a flexible implementation model rather than a rigid pathway. In routine care, interpretation may require repeated review cycles, adjustment for comorbidities and medication changes, reassessment of access or digital barriers, and modification of the plan as patient goals and glucose patterns change. Used in this way, CGM can remain clinically purposeful while limiting overuse, inequity, and unsafe interpretation.

Evidence gaps and future directions

CGM beyond intensive insulin therapy supports selected clinical use, but several practical questions remain unresolved. Current studies and evidence syntheses are strongest for glycemic metrics, including HbA1c, time in range, time above range, time below range, mean glucose, and variability. They provide less certainty about severe hypoglycemia, emergency care, hospitalization, microvascular complications, macrovascular outcomes, mortality, treatment burden, and cost-effectiveness [5-10]. For clinicians and payers, the remaining question is whether useful glucose patterns lead to durable clinical benefit in defined patient groups.

Future research should separate non-insulin-treated, basal insulin-treated, and intensive insulin-treated type 2 diabetes because these groups differ in hypoglycemia risk, medication decisions, and likely mechanisms of benefit. The optimal duration and intensity of use also remain unclear; some patients may need continuous personal CGM, whereas others may need only intermittent or professional CGM.

The behavioral role of CGM requires more precise evaluation. CGM can show patients how meals, activity, medication timing, sleep, illness, and adherence affect glucose patterns, but passive data exposure is unlikely to be enough. Studies should define which education, coaching, clinician review, or digital support makes feedback useful. Patient-centered outcomes should include treatment satisfaction, diabetes distress, food anxiety, self-efficacy, adherence, and burden [10,12].

OTC CGM creates a separate research agenda. FDA-cleared OTC systems provide new access routes for adults not using insulin, but regulatory clearance does not establish long-term clinical effectiveness or safe use in every context [13-15]. Future studies should examine how OTC CGM is used in practice, whether patients discuss results with clinicians, and whether readings lead to appropriate actions, unsafe actions, anxiety, unnecessary restriction, or false reassurance.

Equity research should move from documenting disparities to testing ways to close them. Current evidence shows differences in CGM prescribing by race, ethnicity, insurance status, safety-net setting, and language preference [18,19]. The next step is to evaluate language-concordant education, interpreter-supported training, simplified data sharing, reader-based options, coverage support, community health worker involvement, and team-based review. Current evidence and implementation gaps are most consistent with selective, structured use of CGM beyond intensive insulin therapy, linked to defined clinical or behavioral objectives rather than routine use across all adults with type 2 diabetes.

Limitations

Several features of this review should guide interpretation. This article uses a structured narrative review rather than a systematic review design. Sources were selected to support a focused clinical synthesis of CGM use beyond intensive insulin therapy in type 2 diabetes, covering guidance, regulatory documents, coverage policy, evidence syntheses, selected trials, and equity studies available through March 31, 2026. No protocol-based search, PRISMA screening, risk-of-bias scoring, or quantitative synthesis was performed. The review is intended as a practical clinical framework rather than a complete evidence inventory.

The evidence base is heterogeneous, with studies differing by treatment regimen, device type, wear duration, educational support, comparator, baseline glycemic control, and follow-up. Evidence from non-insulin-treated adults, basal insulin-treated adults, and intensive insulin-treated adults should not be merged into a single conclusion. Current data are strongest for HbA1c and CGM-derived metrics including time in range, time above range, time below range, mean glucose, and glycemic variability. They are less informative about long-term complications, emergency care, hospitalization, mortality, behavior change durability, treatment burden, and cost-effectiveness.

FDA-cleared intended use defines the regulatory basis for marketing and use, but does not prove clinical benefit for every adult with type 2 diabetes or every use case. Medicare coverage policy is an important US reference point, but should not be generalized to commercial insurance, Medicaid, uninsured care, or out-of-pocket OTC purchasing. The included disparities studies address CGM prescribing or orders, not the full pathway to device receipt, sustained use, correct interpretation, patient experience, and outcomes. For these reasons, the review should be interpreted as a practical synthesis supporting selective and structured CGM use, not as a guideline recommending routine device use for all adults with type 2 diabetes.

## Conclusions

CGM is expanding beyond intensive insulin therapy in type 2 diabetes, but wider availability should not be equated with routine use for all patients. In non-insulin-treated adults, its most defensible role is focused pattern recognition and structured feedback when HbA1c or occasional capillary glucose testing cannot answer a management question. In adults treated with basal insulin without prandial insulin, CGM has a clearer role in identifying fasting and overnight patterns, postprandial hyperglycemia, unrecognized hypoglycemia, and possible over- or under-titration. Across these groups, current evidence supports glycemic metrics and clinical interpretation more strongly than long-term outcomes, cost-effectiveness, or universal continuous use.

OTC CGM adds a new access route for selected adults not using insulin, but FDA-cleared device status should be distinguished from proven clinical benefit, prescription CGM, professional CGM, and consumer wellness use. Safe implementation depends on more than the sensor. Patients need education, a clear reason for monitoring, feasible access, language-appropriate support, digital readiness, and guidance on when readings require confirmation or clinician review. Clinicians should interpret CGM with HbA1c, symptoms, medications, comorbidities, hypoglycemia risk, and individualized goals. Used in this way, CGM can function as structured decision support rather than passive surveillance or consumer data collection. The aim is not default continuous monitoring, but selective use of glucose data when they make care safer, more specific, and more actionable.
